# Disparities in accessing specialty behavioral health services during the COVID-19 pandemic and why we need pediatric integrated primary care

**DOI:** 10.3389/fpsyt.2024.1356979

**Published:** 2024-05-10

**Authors:** Ayanda Chakawa, Trista Perez Crawford, Leslee Throckmorton Belzer, Hung-Wen Yeh

**Affiliations:** ^1^ Division of Developmental and Behavioral Health, Section of Pediatric Psychology, Children’s Mercy Hospitals and Clinics, Kansas City, MO, United States; ^2^ University of Missouri Kansas City School of Medicine, Kansas City, MO, United States; ^3^ Emory Pediatric Institute, Emory School of Medicine, Atlanta, GA, United States; ^4^ Children’s Healthcare of Atlanta, Center of Behavioral and Mental Health, Atlanta, GA, United States; ^5^ The Beacon Program, Children’s Mercy Hospitals and Clinics, Kansas City, MO, United States; ^6^ Division of Health Services and Outcomes Research, Children’s Mercy Hospitals and Clinics, Kansas City, MO, United States

**Keywords:** integrated primary care, consultation or warm handoffs, co-located care, specialty behavioral health, access to care, racial disparities

## Abstract

**Objective:**

Youth unmet behavioral health needs are at public health crisis status and have worsened since the onset of the coronavirus disease 2019 pandemic (Covid-19). Integrating behavioral health services into pediatric primary care has shown efficacy in addressing youth behavioral health needs. However, there is limited guidance on facilitating equitable access to care in this setting, including in triaging access to co-located services (i.e., onsite outpatient behavioral health services with only the behavioral health provider) or to specialty behavioral health services in other clinics within larger health systems.

**Methods:**

A retrospective, comparative study was conducted to examine variability in access to co-located and specialty behavioral health (SBH) services for a pre-Covid-19 cohort (April 2019 to March 2020; *n* = 367) and a mid-Covid-19 cohort (April 2020 to March 2021; *n* = 328), while accounting for integrated primary care consultation services. The sample included children 1-18 years old served through a large, inner-city primary care clinic. Logistic regression models were used to examine the association between scheduled and attended co-located and SBH visits, pre- and mid-Covid-19 effects, and sociodemographic factors of race and ethnicity, language, health insurance (SES proxy), age, and sex.

**Results:**

The majority of youth were not directly scheduled for a co-located or SBH visit but the majority of those scheduled attended their visit(s). The odds of not being directly scheduled for a co-located or SBH visit were greater for the mid-Covid-19 cohort, Black youth, and older youth. Accounting for integrated primary care consultation visits addressed these disparities, with the exception of persisting significant differences in scheduled and attended co-located and SBH visits for Black youth even while accounting for IPC consultation.

**Implication:**

Findings from the current study highlight the effective role of integrated primary care consultation services as facilitating access to initial behavioral health services, especially given that referrals to integrated primary care co-located and SBH services within the larger health system often involve barriers to care such as longer wait-times and increased lack of referral follow through. Ongoing research and equitable program development are needed to further this work.

## 1 Introduction

Youth unmet behavioral health needs in the United States (U.S.) are at public health crisis status (1–5) with as low as 29% of children receiving needed care (6). Unmet behavioral health needs are commonly defined as a need for behavioral evaluation or intervention but not using these services within a year (7–10). Among youth this need is generally quantified by the gap between identified behavioral health problems (including by youth, parents, or medical providers) and service utilization (7, 11, 12).

Unmet behavioral health needs are especially pronounced among youth from structurally marginalized sociodemographic backgrounds, including racially and ethnically minoritized^a^ groups (7, 13–16). Research is increasingly examining the effectiveness of integrating behavioral health services into primary care to better meet youth behavioral health needs (17–27). However, there is limited guidance on factors contributing to equitable access to care in this setting, including when needing to triage access to integrated primary care (IPC)^b^ co-located services (i.e., onsite outpatient behavioral health services with only the behavioral health provider (BHP) in the primary care setting) or to specialty behavioral health (SBH) services located in other clinics within larger health systems. Most research has stopped with the acknowledgment that IPC with its various levels of outpatient behavioral health care embedded within the primary care setting can help increase access to care for youth from diverse backgrounds, including those from communities of color^c^, lower socioeconomic status (SES), and/or linguistically diverse groups. Research is starting to explore how the current state of highly integrated IPC service delivery (e.g., IPC consultation) and connection to step-up level services (including co-located care and SBH) may still involve methods that perpetuate inequity (28, 29). These inequities are linked to the lasting historical impact of health care systems developed to meet the needs of majoritized^d^ sociodemographic groups in the U.S while not also centering the needs of minoritized groups (15, 30–32).

### 1.1 IPC consultation in relation to co-located and SBH services

IPC is important to the discussion of addressing disparities in behavioral health care access because behavioral health service-seeking is increasingly taking place in primary care clinics (33, 34). Within primary care, “behavioral health” is a term often used in place of “mental health” to help destigmatize services (17, 20). It is incorporated in the Primary Care Behavioral Health (PCBH) applied theoretical model, which is an evidence-based approach for pediatric IPC service delivery (35–37). Per Reiter et al. (2018), the PCBH model “*incorporates into the primary care team a behavioral health consultant (BHC), sometimes referred to as a behavioral health clinician [or behavioral health provider], to extend and support the primary care provider (PCP) and team.”* The PCBH model recognizes the levels of integration within primary care (e.g., IPC consultation, co-located care), the role of SBH, and triage needs between these levels.

Through the PCBH model, IPC has helped improve the quality of care for youth and their families. However, accessibility and utilization based on sociodemographic status has shown variation by service provision type. More highly integrated IPC service options such as consultation (i.e., joint visits or in-the-moment warm handoffs from the primary care provider (PCP) to the behavioral health provider (BHP)) have been shown to facilitate significantly more timely access, initial, and short-term service utilization (38, 39). However, other levels of service that are typically provided through less directly integrated care or through specialty care options external to the IPC setting are often required when chronic or high acuity concerns are present, or when there is a need for comprehensive evaluation (38, 40). Access to these services is usually initiated through referrals by PCPs or BHPs to step-up level care options such as IPC co-located services or SBH services with a range of other providers (e.g., psychologists, psychiatrists, developmental and behavioral pediatricians, and master’s level therapists or counselors) in other clinics within larger health systems (or potentially through community-based practices or mental/behavioral health centers). Though these services are needed, they frequently result in significantly longer wait-times and increased lack of follow through with service referrals (38), with potentially wider gaps in service utilization for historically underserved groups. Better understanding is needed on whether disparities in access to care exist between these service levels and if so, how best to mitigate the access needs.

### 1.2 Covid-19 pandemic effects on behavioral health care access through IPC

More recently, inequities in behavioral health care access have worsened due to disproportionate effects of the coronavirus disease 2019 pandemic (hereafter referred to as Covid-19). Disparities in access to behavioral health care existed prior to Covid-19, including those related to difficulty coordinating care with specialty providers (41–43). While these factors likely persisted into the pandemic, other challenges were introduced or exacerbated, including increased logistical demands in accessing care as telehealth was more widely introduced to minimize exposure risk to Covid-19 (44). While IPC service provision, especially in pediatric primary care settings, has been a growing initiative to increase behavioral health care access for children (26, 33, 38), its growth intersected with pandemic related changes to the behavioral health landscape. These changes include increased provision of IPC through telehealth mechanisms which have shown some negative impacts on access to care, especially for racially and ethnically minoritized youth with diverse linguistic backgrounds (28, 45). While information on the direct pattern of these effects is still emerging through research, current understanding of the Covid-19 era and its association to behavioral health service access and utilization pre- and mid-Covid-19 is needed, especially in the context of different service levels. This is especially necessary as studies indicate that the behavioral health impacts of Covid-19 (46, 47) and certain service delivery modalities such as telehealth are likely to maintain past the initial and mid-pandemic phases (48, 49).

### 1.3 Race and ethnicity, and accounting for sociodemographic variability in access to care through IPC pathways

Despite the benefits of IPC, research has recently started to acknowledge the complexities of navigating through IPC levels of care and SBH (50) systems, and the resultant racial and ethnic disparities that emerge (34, 38, 51, 52). Even in the adult behavioral health literature there is growing acknowledgment that IPC research has included sizable portions of participants from communities of color but has generally not reported population specific outcomes despite lack of clarity on disparate access to care (53). New insight among pediatric populations, has shown that common approaches to IPC as a strategy to increase behavioral health care access for youth (26, 33) yields some notable racially and ethnically disproportionate gaps in care, including for samples predominantly representing Black and Hispanic/Latin/o/e groups (29, 38, 50, 54, 55) Research further shows that racial and ethnic disparities in access to care widen for patients from communities of color compared to White patients when, in order to receive care, patients must engage in services outside of their primary care visit after the PCP makes the referral to co-located services or outside their medical home when the PCP makes the referral to SBH services (34, 38).

In addition to racial and ethnic group disparities in access to care, other sociodemographic variables such as language, SES (or “health insurance type” as a proxy), age, and sex have been linked to variability in behavioral health care access (56, 57). In particular, youth from groups that represent lower SES background, racial or ethnic minoritization, family preference for another language other than English for health care needs, female (compared to male), and younger age ranges have shown more stability in low rates of access to behavioral health care (16, 51, 58). However, the pattern of these effects is less clear in the primary care setting but necessitates ongoing monitoring.

### 1.4 The current study

This study employed a two-cohort comparative, retrospective design based on two groups, a pre-Covid-19 cohort and a mid-Covid-19 cohort to explore access to needed co-located and SBH services across racial and ethnic groups after referral through pediatric primary care by a PCP. The purpose of the study was to increase understanding of accessibility to these services while accounting for unique contributions of IPC consultation in facilitating service access through integrated primary care, and accounting for other sociodemographic factors of socioeconomic background, and preferred language for health care services. Based on the review of the literature as outlined in the main sections above, it is expected that:

1. The majority of youth referred for co-located and SBH services overall will not get directly scheduled for the co-located or SBH service they were referred to but of those scheduled, the majority will attend their scheduled visit;
2. The mid-Covid-19 cohort will schedule and attend co-located and SBH services at a lower rate than the pre-Covid-19 cohort but this difference will be mitigated when accounting for utilization of IPC consultation services; and
3. Youth from communities of color (compared to White youth) will access services at a significantly lower rate, especially among the mid-Covid-19 cohort. Specific hypotheses about the other sociodemographic variables are not made *a priori* due to limited existing research in this area for the pediatric IPC setting.

## 2 Materials and methods

### 2.1 Sample

The cohorts for the current study were defined as the pre-Covid-19 cohort (April 2019 to March 2020; *N* = 367) and the mid-Covid-19 cohort (April 2020 to March 2021; *N* = 328). Both cohorts consisted of patients aged 1-18 years old who were referred for IPC co-located or SBH services by their PCP. The focal pediatric primary care clinic for the study is a large, inner-city Patient-Centered Medical Home (PCMH) that is part of a large, regional children’s hospital in a moderate-sized metropolitan city in the Midwest region of the U.S. The patients and families served through the pediatric primary care clinic represent an ~83% Medicaid patient population, diverse backgrounds (86% from communities of color, 28% preference for health care services in a language other than English, and 52% female) and an overall population with substantial psychosocial, socioeconomic, and cultural barriers. Demographic characteristics specific to the study sample are presented in **Table 1**.

**Table 1:** Sample demographics and key variables for the overall sample and grouped by scheduled and attended co-located and specialty behavioral health visits accounting for integrated primary care (IPC) consultation visits.

Variables	Full Sample	Scheduled (Not accounting for IPC Consultation)		Scheduled (Accounting for IPC Consultation)		Attended (Not accounting for IPC Consultation)		Attended (Accounting for IPC Consultation)	
		No	Yes	No	Yes	No	Yes	No	Yes
Cohort *n* (%)									
Pre-Covid	367 (52.8)	220 (49.8)	147 (58.1)	195 (52.1)	172 (53.6)	17 (63.0)	130 (57.5)	20 (54.1)	152 (53.5)
Mid-Covid	328 (47.2)	222 (50.2)	106 (41.9)	179 (47.9)	149 (46.4)	10 (37.0)	96 (42.5)	17 (45.9)	132 (46.5)
Race/Ethnicity *n* (%)									
White	189 (27.2)	105 (23.8)	84 (33.2)	91 (24.3)	98 (30.5)	7 (25.9)	77 (34.1)	8 (21.6)	90 (31.7)
Black	230 (33.1)	157 (35.5)	73 (28.9)	135 (36.1)	95 (29.6)	12 (44.4)	61 (27.0)	19 (51.4)	76 (26.8)
Hispanic/Latin/o/e	176 (25.3)	123 (27.8)	53 (20.9)	101 (27.0)	75 (23.4)	5 (18.5)	48 (21.2)	6 (16.2)	69 (24.3)
Other	100 (14.3)	57 (12.9)	43 (17.0)	47 (12.6)	53 (16.5)	3 (11.1)	40 (17.7)	4 (10.8)	49 (17.3)
Language for Healthcare Needs *n* (%)									
English	562 (80.9)	348 (78.7)	214 (84.6)	299 (79.9)	263 (81.9)	23 (85.2)	191 (84.5)	31 (83.8)	232 (81.7)
Spanish	115 (16.5)	83 (18.8)	32 (12.6)	66 (17.6)	49 (15.3)	4 (14.8)	28 (12.4)	5 (13.5)	44 (15.5)
Other	18 (2.6)	11 (2.5)	7 (2.8)	9 (2.4)	9 (2.8)	0 (0.0)	7 (3.1)	1 (2.7)	8 (2.8)
Health Insurance Type *n* (%)									
Private	120 (17.3)	68 (15.4)	52 (20.6)	61 (16.3)	59 (18.4)	3 (11.1)	49 (21.7)	3 (8.1)	56 (19.7)
Medicaid/Self-Pay	575 (82.7)	52 (20.6)	201 (79.4)	313 (83.7)	262 (81.6)	24 (88.9)	177 (78.3)	34 (91.9)	228 (80.3)
Age *M* (*SD*)	7.5 (4.0)	7.8 (4.2)	7.1 (3.8)	7.7 (4.2)	7.3 (3.8)	6.9 (3.4)	7.1 (3.8)	7.1 (3.5)	7.3 (3.9)
Sex *n* (%)									
Male	457 (65.8)	287 (64.9)	170 (67.2)	241 (64.4)	216 (67.3)	18 (66.7)	152 (67.3)	27 (73.0)	189 (66.5)
Female	238 (34.2)	155 (35.1)	83 (32.8)	105 (32.7)	105 (32.7)	9 (33.0)	74 (33.7)	10 (27.0)	95 (33.5)

N = 321-695.

### 2.2 Focal behavioral health services

The following focal behavioral health services form the tiers of care initially stemming from the IPC setting and broadening to SBH services within the hospital system.

#### 2.2.1 IPC consultation

IPC consultation services were based on ~30-minute joint appointments with a PCP, psychologist, psychology resident, or psychology intern. The consultations were provided during PCP Well Child and Ill visits, based on PCP or patient/family request for an appointment to address a behavioral health need. The consults were scheduled into three to five pre-defined time slots during half-day clinics and were visible to all providers. Scheduling was completed by nursing or administrative staff either while a patient was in-clinic for a medical visit or if caregivers contacted the clinic to request support for their child’s behavioral health need. Consultation visits were typically scheduled prior to a clinic session. Live/unscheduled consults (i.e., in-the-moment warm handoffs) could be added if the scheduled time slots were not full, though capacity for add-ons was limited.

The consultation service was developed to provide expedient access to care and targeted intervention for patients with milder psychological, behavioral, and emotional concerns and then triage patients to ongoing needed services if clinically indicated. During IPC consultation, the psychology team assessed the child or adolescent for conditions such as Attention-Deficit/Hyperactivity Disorder (ADHD), anxiety, depression, trauma, school concerns, questions of autism, and suicidal ideation. The team then provided a targeted intervention plan, recommendations, and collaborated with the PCP to determine follow-up either through another IPC consultation, co-located psychology services, or SBH services in the hospital or community clinics. Pre-Covid-19, the psychology team worked on-site, in the clinic, and provided services in the medical rooms and a designated therapy room in the clinic. Mid-Covid-19, the psychology team started providing scheduled telehealth IPC consultation services approximately two months into the pandemic, through joint telehealth visits with PCPs.

#### 2.2.2 IPC co-location

The co-located services in the current study include Primary Care Clinic (PCC) Psychology and the ADHD Clinic.

PCC Psychology received electronic referrals from PCPs for common presenting concerns of ADHD, anxiety, depression, school concerns, trauma, etc. Directly referred patients were contacted to confirm interest in services and then sent an intake packet (available in Spanish or English, with interpretation-service options available for other languages). If a patient was not reached during two attempted phone calls (spaced one week apart and including voicemails with a direct callback number), a letter requesting the patient call to schedule was sent to the address on file in the EMR. Intake packets included a caregiver-completed form to describe presenting problems and broadband behavioral rating scales. Returned packets were reviewed and then patients were triaged based on presenting problem to services within the co-location model or to a specialty service within another hospital-based clinic (e.g., Developmental & Behavioral Health Clinic services, ADHD Clinic). The waitlist period was 3-8 months depending on the service demand, and then caregivers had to respond to clinic efforts to reach them to schedule their appointment. These appointments were scheduled 2-4 weeks out from the scheduling call.

Two main services were provided through the PCC Psychology IPC co-location: (1) evidence-based therapy and (2) comprehensive evaluation. Evidenced-based brief intervention (three to five sessions) or time-limited therapy (average of ten to twelve sessions) included outpatient psychotherapy (e.g., behavioral parent training, school consultation/planning, emotion regulation/coping skill development) and collaboration with the PCP (more frequent for patients on medication) regarding treatment plan and progress. Pre-Covid-19, the co-located visits were conducted in-person in the clinic. Mid-Covid-19, the co-located appointments were conducted via telehealth.

The ADHD Clinic received PCP referrals for children who needed specialty medicinal and behavioral health follow-up for ADHD. As part of the intake process, caregivers had to respond to clinic efforts to reach them to confirm their mailing address, and then receive and complete an intake packet with questionnaires for parent and teacher completion. The packet materials were available in English and Spanish and when necessary, were completed over the phone or in person with interpreters in other languages (e.g., French, Swahili, etc.). The caregiver had to return the materials to the clinic, for the child to be placed on the clinic waitlist. The caregiver then had to respond to scheduling calls from the clinic after the ~3-4 month waitlist period to confirm an appointment date and time that would be scheduled 2-4 weeks out from the scheduling call.

For the focal time points of this study, the ADHD Clinic was an interdisciplinary program designed to treat symptoms of complex ADHD in children 3 years of age through adolescence. It consisted of a physician, psychologist, licensed clinical social worker, care assistant and registered nurse. The clinic provided medication management and behavioral interventions in the form of joint behavioral and medication consultation visits, parent behavior management groups, and individual medication and behavioral therapy appointments. Pre-Covid-19, the visits were scheduled “in person” at the same building as the primary care clinics. Mid-Covid-19, the clinic solely offered telehealth appointments. Patients were brought into the clinic for periodic weight checks and blood draws as needed.

#### 2.2.3 SBH

The SBH services included in the current study were provided through the Developmental & Behavioral Health (DBH) Clinic. The DBH Clinic received direct PCP referrals for children who needed specialty behavioral and developmental health evaluations or intervention services. For the study focal period, to access DBH Clinic services after a PCP referral, families from the hospital-based primary care clinics had to call the DBH Clinic to express interest in receiving services, complete an intake over the phone or by email, and then wait for approval to receive services based on the intake data. After approval for a specific service, families could expect to experience between one to all of the following steps depending on the protocol for the specific service referral: receive and complete a new patient questionnaire with forms for parent and teacher completion (provided in English or Spanish, with interpretation-service options available for other languages), return the new patient questionnaire packet in order to be placed on the clinic waitlist, and then respond to scheduling calls from the clinic after the ~3-11 month waitlist period (length depending on the service) to confirm an appointment date and time that would be scheduled 2-4 weeks out from the scheduling call.

The DBH Clinic was located offsite from the primary care clinics, at the larger pediatric hospital. Its outpatient evaluation and intervention services were provided through the Autism Team, General Clinical Child Team, and specialty medical teams, including Developmental Pediatrics and Psychiatry. Pre-Covid-19, patients were scheduled to be seen “in person” in the clinic. Mid-Covid-19, visits were scheduled via telehealth and “in person.” Diagnostic Interviews and interpretation sessions were often scheduled via telehealth. Testing sessions were scheduled “in person” when possible. Therapy sessions were scheduled “in person” and via telehealth.

### 2.3 Procedures

#### 2.3.1 Data extraction

This study was approved by the hospital’s Institutional Review Board (IRB) for human subjects research. A retrospective records review of the electronic medical record (EMR) was conducted to gather data on patients referred for IPC co-located or SBH services between April 2019 to March 2020 (pre-Covid-19 cohort) and April 2020 to March 2021 (mid-Covid-19 cohort). All data were accessed and stored in Health Insurance Portability and Accountability Act (HIPAA) compliant institutional data management databases and deidentified for data processing.

### 2.4 Measures

#### 2.4.1 Dependent variables

##### 2.4.1.1 Scheduled (not accounting for IPC consultation)

The *scheduled (not accounting for IPC consultation)* variable was used as an output variable to measure direct service access to co-located and SBH services, without accounting for whether a patient received IPC consultation services within the cohort year. It consisted of two categories (“no” = 0 and “yes” = 1) to assess if at least one directly scheduled co-located or SBH service was scheduled within a year of that service referral, regardless of whether IPC Consultation was conducted. The “yes” categorization was fulfilled for this variable only if a participant had scheduled a co-located or SBH visit within a year after the referral.

##### 2.4.1.2 Scheduled (accounting for IPC consultation)

The *scheduled (accounting for IPC consultation)* variable was used as an output variable to measure direct service access to co-located and SBH services, while also accounting for whether a patient received IPC consultation services. This variable consisted of two categories (“no” = 0 and “yes” = 1) to assess if an initial behavioral health care visit for at least one of these three visit types was scheduled within a year of the co-located or SBH service referral. The “yes” categorization was fulfilled for this variable if a participant had scheduled a co-located or SBH visit, and/or completed an IPC consultation within a year after their co-located or SBH service referral.

##### 2.4.1.3 Attended (not accounting for IPC consultation)

The *attended (not accounting for IPC consultation)* variable was used as an output variable to measure direct service access to co-located and SBH services, without accounting for whether a patient received IPC consultation services within the cohort year. It consisted of two categories (“no” = 0 and “yes” = 1) to assess if at least one directly scheduled co-located or SBH service was attended within a year of that service referral. The “yes” categorization was fulfilled for this variable only if a participant had attended a co-located or SBH visit within a year after the referral.

##### 2.4.1.4 Attended (accounting for IPC consultation)

The *attended (accounting for IPC consultation)* variable was used as an output variable to measure direct service access to co-located and SBH services, while also accounting for whether a patient received IPC consultation services. This variable consisted of two categories (“no” = 0 and “yes” = 1) to assess if a behavioral health care visit for at least one of these three visit types was attended within a year of the co-located or SBH service referral. The “yes” categorization was fulfilled for this variable if a participant had attended a co-located or SBH visit, and/or completed an IPC consultation within a year after their co-located or SBH service referral.

#### 2.4.2 Predictor variables

##### 2.4.2.1 Cohort

The *cohort* variable was used to define cohorts for those who were referred for behavioral health services between April 2019 to March 2020 (“pre-Covid” = 0) and April 2020 to March 2021 (“mid-Covid” = 1).

##### 2.4.2.2 Race and ethnicity

A four-category variable was constructed to represent *race and ethnicity:* 0 = “White,” 1 = “Black,” 2 = “Hispanic/Latin/o/e,” and 3 = “Other”. The “Other” category is comprised of racial and ethnic groups with small subsample sizes (Asian, Native American, Multiracial, and Native Hawaiian or Pacific Islander groups) that would preclude meaningful statistical sample sizes if left as standalone groups.

#### 2.4.3 Covariates

##### 2.4.3.1 Preferred language for health care

A three-category variable was constructed to represent *preferred language for health care*: 0 = English, 1 = Spanish, and 2 = Other (including preferred languages of Somali, Amharic, Arabic, Bengali, Farsi, Sign Language, and Vietnamese). Similar to the race and ethnicity variable, the languages in the “Other” category were not highly represented in the sample so were collapsed to form an overall group.

##### 2.4.3.2 Health insurance type

The *health insurance type* variable is used as a proxy for socioeconomic status (SES). It was dichotomized (0 = “Commercial/Private” and 1 = “Medicaid/Self-Pay”) to address low group frequency counts and to foster meaningful data comparisons. At the focal primary care clinic, children with “Self-Pay” status are likely to get financial assistance from the hospital to cover or subsidize medical bills similar to coverage considerations for children with Medicaid, as they usually do not have private insurance coverage that is typically reflective of family employment or wealth differences.

##### 2.4.3.3 Age

Patient *age* was included as a continuous variable defined by how many years old a child was at the time of the co-located or SBH service referral.

##### 2.4.3.4 Sex

A dichotomous variable for patient *sex* was used (“male” = 0 and “female” = 1).

#### 2.4.4 Data analytical design

Descriptive analyses were used to explore category frequencies across all variables. For the primary analysis, a series of binomial logistic regression models were conducted to examine the association between the four dependent variables “*scheduled (not accounting for IPC consultation),” “scheduled (accounting for IPC consultation),” “attended (not accounting for IPC consultation),” and “attended (accounting for IPC consultation)”* and the predictor variables “*cohort*” and “*race and ethnicity*.” All models controlled for potential covarying effects of “*preferred language for health care*,” “*health insurance type*,” “*age*,” and “*sex.*”

Descriptive statistic exploration and the binomial logistic regression analyses for the overall sample were conducted using the Statistical Package for the Social Sciences (SPSS), Version 24 (59). A *p* value of ≤.05 was used to determine statistical significance. Propensity score matching based on the optimal full matching was conducted in follow up analyses using *R, Version 4.3.1.* (60) using the MatchIt package version 4.5.5 (61). Propensity score matching was used to evaluate the marginal effect of *cohort* and *race and ethnicity* on the *scheduled* and *attended* variables, isolating the potential confounding factors of the other included covariates.

## 3 Results

### 3.1 Descriptive data

The preliminary descriptive data representing the study variables showed that rates of scheduled (pre-Covid-19 *n* = 147 (40.1%); mid-Covid-19 *n* = 106 (32.3%)) and attended (pre-Covid-19 *n* = 130 (35.4%); mid-Covid-19 *n* = 96 (29.3%)) co-located and SBH visits were low overall, but especially for visits attended mid-Covid-19. However, the majority of youth directly scheduled for a co-located or SBH visit attended their visit (see **Table 1**). There was an overall pattern of increased service access frequency when IPC consultation was accounted for between the dependent variables *scheduled (not accounting for IPC consultation)* vs. *scheduled (accounting for IPC consultation)* and *attended (not accounting for IPC consultation)* vs. *attended (accounting for IPC consultation*; see **Table 1**). This pattern was found between cohorts and across the sociodemographic variables. Additionally, the ratio of youth from communities of color (*n* = 506 (72.8%)) compared to White youth (*n* = 189 (27.2%)) in the overall sample underrepresented the general clinic racial distribution of ~86% of patients from communities of color. The ratio of females compared to males was also underrepresented (*n* = 238 (34.2%) vs. *n* = 457 (65.8%), respectively, compared to the general clinic distribution of 52% female patients. Similarly, patients who preferred health care services in a language other than English were also under-represented in the full study sample (*n* = 133 (19.1%)) compared to the general clinic distribution of those who had a preference for health care services in a language other than English (~28%).

### 3.2 Primary data analyses

A series of binomial logistic regression models (see **Table 2**) were used to explore variability in service access based on associations between the four dependent variables (scheduled (not accounting for IPC consultation), scheduled (accounting for IPC consultation), attended (not accounting for IPC consultation), and attended (accounting for IPC consultation)) and the predictor variables cohort and race and ethnicity, while accounting for potential covarying effects of preferred language for health care, health insurance type, age, and sex. Across all the models, the Hosmer–Lemeshow tests were not statistically significant (χ^2^s[8] = 4.618 – 10.395, *p*s = .238 -.798), indicating goodness-of-fit. The omnibus test of model coefficients was only statistically significant (χ^2^[8] = 25.432, *p* <.001) for the model examining associations between scheduled (not accounting for IPC consultation) co-located and SBH visits, indicating improvement in model accuracy with the included predictor variable and covariates. This model also correctly classified 63.0% of cases and explained 4.9% of the variance (per Nagelkerke *R* ^2^) between the variables. The omnibus test of model coefficients was not statistically significant (χ^2^[8] = 5.875 – 12.880, *p* = .116 -.661) for the remaining models, indicating no improvement in model accuracy with the predictor variable and covariates. These remaining models correctly classified 54.8 - 89.3% of cases and explained 1.9 – 7.7% of the variance (per Nagelkerke *R* ^2^) between the variables.

**Table 2:** Binomial logistic regression models examining variability in scheduled and attended co-located and specialty behavioral health visits with and without accounting for integrated primary care (IPC) consultation visits.

Variables	*B (S.E.)*	*p*	Odds Ratio (95% CI)
Scheduled (not accounting for IPC Consultation)			
Cohort	-0.371 (.167)	.024	0.685 (0.493, 0.951)
Race/Ethnicity			
White vs. Black	-0.541 (.206)	.009	0.582 (0.389, 0.872)
White vs. Hispanic/Latin/o/e	-0.415 (.277)	.134	0.661 (0.384, 1.136)
White vs. Other	-0.066 (.253)	.794	0.936 (0.571, 1.538)
Language for Healthcare Needs	-0.038 (.173)	.824	0.962 (0.686, 1.350)
Health Insurance Type	-0.346 (.211)	.100	0.707 (0.468, 1.069)
Age	-0.056 (.021)	.009	0.946 (0.907, 0.986)
Sex	-0.038 (.173)	.824	0.962 (0.686, 1.350)
Scheduled (accounting for IPC Consultation)			
Cohort	-0.061 (.159)	.701	0.941 (0.689, 1.284)
Race/Ethnicity			
White vs. Black	-0.427 (.199)	.032	0.653 (0.442, 0.963)
White vs. Hispanic/Latin/o/e	-0.355 (.265)	.180	0.701 (0.418, 1.178)
White vs. Other	0.029 (.250)	.908	1.029 (0.631, 1.680)
Language for Healthcare Needs	0.018 (.258)	.945	1.018 (0.614, 1.688)
Health Insurance Type	-0.156 (.206)	.450	0.856 (0.572, 1.281)
Age	-0.026 (.020)	.195	0.975 (0.938, 1.013)
Sex	-0.097 (.164)	.557	0.908 (0.658, 1.253)
Attended (not accounting for IPC Consultation)			
Cohort	-0.278 (.452)	.538	1.321 (0.545, 3.204)
Race/Ethnicity			
White vs. Black	-0.721 (.511)	.865	0.877 (0.194, 3.975)
White vs. Hispanic/Latin/o/e	-0.131 (.771)	.692	1.332 (0.322, 5.506)
White vs. Other	0.287 (.724)	.692	1.332 (0.322, 5.506)
Language for Healthcare Needs	0.051 (.768)	.947	1.053 (0.234, 4.743)
Health Insurance Type	-0.811 (.645)	.209	0.445 (0.126, 1.573)
Age	0.029 (.059)	.617	1.030 (0.918, 1.155)
Sex	-0.166 (.449)	.712	0.847 (0.351, 2.044)
Attended (accounting for IPC Consultation)			
Cohort	0.086 (.373)	.818	1.089 (0.525, 2.261)
Race/Ethnicity			
White vs. Black	-0.982 (.453)	.030	0.375 (0.154, 0.911)
White vs. Hispanic/Latin/o/e	0.221 (.716)	.758	1.247 (0.306, 5.076)
White vs. Other	0.182 (.642)	.777	1.199 (0.340, 4.225)
Language for Healthcare Needs	-0.176 (.647)	.786	0.839 (0.236, 2.979)
Health Insurance Type	-1.055 (.634)	.096	0.348 (0.101, 1.205)
Age	0.016 (.049)	.749	1.016 (0.923, 1.118)
Sex	0.203 (.407)	.618	1.225 (0.552, 2.721)

N = 253-695. Reference category is “0” for all variables. Cohort is coded as 0 = “Pre-Covid” and 1 = “Mid-Covid.” Race/Ethnicity is coded as 0 = “White,” 1 = “Black,” “2 = “Hispanic/Latin/o/e,” and 3 = “Other” (including Asian, Native American, Multiracial, and Native Hawaiian or Pacific Islander groups). Preferred Language for Health Care is coded as 0 = “English” and 1 = “Other.” Health Insurance Type is coded 0 = Commercial/Private and “1” = Medicaid/Self-Pay. Age is a continuous variable. Sex is coded as 0 = “Male” and 1 = “Female.” The dependent variables Scheduled (not accounting for IPC Consultation), Scheduled (accounting for IPC Consultation), Attended (not accounting for IPC Consultation), and Attended (accounting for IPC Consultation) were coded as 0 = “No” and 1 = “Yes.”

When examining the frequency of patients who were directly scheduled for co-located and SBH visits compared to those who were not directly scheduled after PCP referral for one of these services (i.e., not transitioned to IPC co-located services through IPC consultation services), significant associations were found across cohorts and by race and ethnicity when IPC consultation was not accounted for. Specifically, the odds of the mid-Covid-19 cohort being scheduled was 0.69 times less compared to the pre-Covid-19 cohort. Also, Black youth were 0.58 times less likely to be scheduled compared to White youth. Statistically significant covariate effects in directly scheduled co-located and SBH visits without accounting for IPC consultation were found in relation to age. Older youth were .95 times less likely to be scheduled compared to younger youth when not accounting for IPC consultation services. When accounting for IPC consultation, despite the increase in access to services across all racial and ethnic groups, the pattern of Black youth being significantly less likely to be scheduled for co-located or SBH visits compared to White youth persisted but cohort- and age-related effects did not.

When examining the frequency of patients who attended co-located and SBH visits that were directly scheduled compared to those who did not attend after PCP referral for one of these services, no significant cohort or sociodemographic associations were found when IPC consultation was not accounted for. However, when IPC consultation was accounted for, despite the increase in service access across all groups, the odds of Black youth attending services overall was significant at .04 times lower compared to White youth.

The other covariate variables (health insurance type, preferred language for health care, and sex) were not significantly associated with service access when the effects of the other variables were accounted for across the models. Additionally, the inclusion of the other covariates had nearly no impacts on the effects of cohort and race and ethnicity on scheduling and attendance.

### 3.3 Follow-up analyses

Applying propensity score matching on each exposure and outcome variable led to negligible differences in numerical values (OR +/- 0.02) and resulted in similar *p*-values. This finding was anticipated given the small number of covariates and their weak associations with the outcome variables as suggested by Akaike Information Criterion from comparing the models with and without the covariates.

## 4 Discussion

Gaps in the health care system level help-seeking pathway were present prior to Covid-19 but have been exacerbated by pandemic effects which have disproportionately impacted youth from communities of color (22, 41, 44). The current study adds critical new information on the role of IPC in helping to meet health care system level access to behavioral health care needs for patients served through primary care. It also highlights current gaps that may continue to widen disparate access to care, with the potential of detrimental long-term impact for traditionally underserved youth, especially those from racially and ethnically minoritized backgrounds. Innovative, culturally grounded approaches to IPC are needed to address current behavioral health access disparities.

### 4.1 Tiers of care initially stemming from IPC in relation to access to care

As hypothesized, the majority of youth referred for co-located and SBH services overall were not directly scheduled for those visits, but of those directly scheduled, the majority attended their scheduled visit. This indicates that once families successfully navigate through IPC co-located or SBH service-seeking pathways and are scheduled for an appointment, they are likely to attend that appointment.

While co-located and SBH services are often necessary step-up levels of care due to comprehensive assessment or longer-term intervention needs, limited access in relation to need for services raises concern for the long-term impact of delayed access to care. In addition to the pandemic effects further discussed below, the complexities in behavioral health care access are also likely exacerbated by pediatric patients and their families often being left on their own to navigate behavioral health systems. As the services in these systems become less integrated, they in turn require an increased number of resources, time, persistence, planning, and organization in order to access them. Many families have competing responsibilities that absorb the time necessary to execute a complex system from start to finish, especially if it requires multiple steps with added barriers such as mail delivery for paper forms or extensive questionnaires that have to be returned before a patient can be placed on a waitlist or scheduled for an initial visit. Development of more effective and more accessible systems of behavioral health care is needed, along with use of family navigation support to help address these barriers – even in the IPC setting – to help families get connected to the next tier of care when clinically indicated (50). There are strategies that can also be used from the initial point of IPC consultation to help support better access to SBH, as well as co-located care. These include having SBH intake or new patient forms available in the primary care clinic for families to get intake processes started right away, with the opportunity to ask questions from providers or clinic staff about what is needed for completion of the paperwork.

Research evidence is increasingly supporting the expansion of models of IPC as evidence-based practice to increase access to behavioral health care (37, 54, 62) but methods on how to best do this are still emerging. Our IPC consultation and co-location services were developed to increase access to care by housing psychological services in the primary care location, reducing wait times for services through the opportunity to meet with a psychology provider during medical visits (same-day or within 1-2 weeks), and guided intake procedures with shorter waitlists (1-2 months) for separate, psychology-only visits. However, disparities in service access, including those that emerge when IPC consultation is limited to scheduled and/or telehealth options and when IPC co-located care uses traditional intake paperwork processes and waitlist structures, highlight the need to understand and tailor IPC access within specific populations, while maintaining evidence-based tenets of the approach (i.e., increasing access to effective and equitable care). Extensions of IPC models that elevate equity and access while minimizing barriers could do well to embed more specialty services into the PCMH (e.g., ADHD Clinics with robust behavioral intervention for children and adolescents, targeted ASD evaluation components, etc.) and walk-in services in primary care with more direct triage pathways that minimize barriers (e.g., reduce intake questionnaire packets or forms required prior to being seen).

### 4.2 Covid-19 effects and sociodemographic factors as unique but critical considerations in IPC and beyond

#### 4.2.1 Covid-19 pandemic effects

Also as hypothesized, the mid-Covid-19 cohort was directly scheduled for co-located and SBH services at a lower rate than the pre-Covid-19 cohort, but this difference was mitigated (made no longer clinically significant) when accounting for initial service access through IPC consultation. Specifically, of the patients who did not access recommended co-located or SBH services within a year of service referral, many from each cohort received some level of behavioral health care through IPC consultation in a way that reduced significant differences in service access pre-Covid-19 and mid-Covid-19. This suggests that IPC consultation was an effective stopgap measure to address lower rates of access to the less integrated (co-located services) and more traditional (SBH services) means of behavioral health care during the Covid-19 pandemic. There were no cohort-based differences in attending directly scheduled IPC co-located and SBH visits.

Though the Covid-19 pandemic response in the care setting was managed with best intentions for safety of patients and staff, we are now better able to determine the impact of the pandemic on children and families’ service access as we have transitioned to the Covid-19 endemic stage. Some mid-Covid-19 service access impacts may have been due to situational pandemic factors (e.g., exacerbated demands related to childcare, work, or illness) that contributed to challenges completing intake or scheduling processes. Also, of note, the mid-Covid-19 cohort in the current sample largely received IPC consultation through telehealth mechanisms. Our previous research on this population highlighted the disparities in service access during the initial year of the pandemic (28), with the current study extending the previous IPC consultation specific findings to disparities pre-Covid-19 compared to mid-Covid-19 in getting a directly scheduled IPC co-located or SBH visit. That is, the telehealth modality did not serve all families equitably, despite best efforts to hold visits safely. As noted in other studies (28, 48, 63–65), access to care mid-Covid-19 may also have been impacted by barriers to telehealth service delivery that can relate to families’ willingness to utilize telehealth services - including technology demands, perceived depersonalization of visits conducted via telehealth, and concern about distractions in the home environment contributing to less effective visits. These barriers necessitate system- and community-based mitigation strategies, including use of easy-access telehealth platforms, technology support at an appropriate literacy level and in a language preferred for healthcare needs prior to and during visits, and helping families access telehealth devices and internet options for free or at reduced costs through local community resources (64, 65). When fully in-person behavioral health visit options are not available for those with telehealth barriers, having in-person hybrid visits (i.e., in-clinic care staff connecting the family in the clinic to the behavioral health provider remotely) should also be considered instead of exclusively remote or telehealth-only options.

Within and outside of the Covid-19 pandemic years, highly integrated behavioral health services helped increase partnerships across PCPs, BHPs, and patients/caregivers, making the consultation or warm handoff approach an effective modality of IPC (29, 53, 55). In particular, IPC consultation provides a practical and timely opportunity for innovating care that results in greater behavioral health service access, as shown in the current study by the Covid-19 pandemic related effects on scheduled co-located and SBH visits being mitigated by IPC consultation access. On the other hand, co-located care often models after traditional approaches to mental health care and in some clinic settings due to requiring processes that become non-starters for families who may not want to navigate factors that may be included with the service. These factors could include wait times, intake or new patient paperwork, or “referral” related wording that can contribute to patients perceiving that they are receiving a different type of service that is not part of the PCMH. In contrast, some populations may prefer to utilize co-located care because they rely on the predictability or organization of scheduled visits, or the ability to access follow up psychotherapy services in a longer term or more traditional manner.

#### 4.2.2 Race and ethnicity and other sociodemographic factors

As hypothesized, racial and ethnic group differences were significant, with Black youth being scheduled for co-located and SBH at a significantly lower rate than White youth. Interestingly, significant racial and ethnic group differences were not observed when exploring visits attended from direct scheduling of IPC consultation and SBH visits but were observed when accounting for IPC consultation visits. Age related differences were also found in relation to older youth being less likely than younger youth to be scheduled through direct referral for co-located and SBH service, only when not accounting for IPC consultation services since consultation closed the significant gap in the age differences. This finding is consistent with research that embedded IPC helps address access to care across the youth age span (66). No other significant sociodemographic group differences were found, though the represented ratio of linguistically diverse, female, and overall youth from racially and ethnically minoritized backgrounds was descriptively low compared to the overall clinic ratios.

Though IPC has helped increase access to evidence-based behavioral health care (17, 20), including for patients from sociodemographically diverse backgrounds (28, 38, 54) disparities in service access still exist (7, 13). Similar to our previous work (28), Black youth in particular from this population were observed to experience more service access disparities compared to White youth, though access to care across racially and ethnically minoritized groups was descriptively low overall – especially based on the clinic population racial and ethnic demographics. Multiple psychosocial factors contribute to inequities in access to behavioral health care for traditionally underserved and marginalized groups. In particular, racial and ethnic disparities in behavioral health care access are associated with cultural and systemic factors, including barriers to care (e.g., stigma) and low overall service readiness/motivation that often stem from a lack of culturally sensitive interventions and a history in the U.S. of inequitable treatment and outcomes in health care (67–69).

IPC is needed and is uniquely positioned as a way to increase access to care, but it is necessary to consider who has been best served within traditional methods of IPC and what methods work best for given populations. IPC research must include population specific outcomes to ensure that this model of behavioral health service provision is increasingly receiving focus and effort in establishing and developing systems that are equitable. It is easy to perceive approaches are doing well if patients are utilizing services and providers are productive, but we must also examine who is being served within clinic populations. Cultural tailoring of initiatives is needed to clarify how diverse patient families will interact with varying levels of IPC and SBH (including service referrals) in order to equitably serve youth from communities of color, especially those from lower SES and linguistically diverse backgrounds (29, 38, 54, 55). For some families, including those from Black racial and ethnic backgrounds, starting with IPC consultation may help build trust toward BHPs, reduce stigma, and increase buy-in for other tiers of behavioral health services. BHPs collaborating with PCPs to address potential sociodemographic disparities occuring from the point of referral may also be needed, as access to care disparities may start at initial stages of the referral process as potentially indicated in the current study based on some disproportionality in the clinic demographics compared to the referral demographics. Also, previous research shows that during Covid-19, HIPAA compliant chat or text behavioral telehealth was used at higher rate by racially and ethnically minoritized youth, and that Black youth were less likely to utilize in-person visits compared to White youth (70). Innovative text-based communication/chat modalities warrant further evaluation as a culturally responsive behavioral telehealth strategy to increase access to care in IPC and SBH settings.

Pre-Covid-19, IPC consultation was conducted both through scheduled joint visits with medical providers and live in-the-moment paged consultations. Mid-Covid-19, this changed abruptly to IPC consultation delivered via telehealth, which eliminated the live-in-the moment paging option during that period. Importantly, the significant racial and ethnic group inequities observed in the current study in relation to IPC consultation are likely inextricably linked to the modality change (i.e., telehealth) and removal of access to real-time behavioral health consultation in primary care. Recent research demonstrates that more structurally and socially vulnerable living areas have lower broadband capabilities which inhibits features necessary to utilize telehealth services; thus, addressing this is essential to improve equitable telehealth care access (71). Understanding of how minoritized groups experience behavioral telehealth care across various contexts as either a facilitator to services (e.g., introducing services that were not previously available) or an inhibitor to services (e.g., limiting access to previously accessible in-person services) based on lessons learned from previous research is needed to inform responsive program development strategies across service levels.

Prior research shows that behavioral health needs among U.S. youth have been a long-standing public health concern (3, 6), especially for youth from communities of color (55). In particular, Black pediatric patients specifically face under-recognition of mental health disorders during the first step to identifying behavioral health needs, thus subsequently impacting the care they receive (72, 73). Culturally responsive efforts that can be scaled and generalized across the U.S. to address growing behavioral health disparities between and within sociodemographic groups are needed (72, 74–77). Examples include leveraging lay/community health workers to assist with behavioral health care navigation needs and going beyond ‘clinic walls’ to establish partnerships with racially and ethnically diverse local community-based settings (e.g., churches, schools, or community centers that serve and engage families from communities of color). These partnerships may include embedding access to free broadband or Wi-Fi services, computers, or smart devices; creating IPC telehealth satellite or multi-site hybrid visit modalities; and facilitating behavioral health service stigma reduction initiatives.

### 4.3 Limitations

Our study has some limitations that should be considered when interpreting our findings. First, technical difficulties known to the telehealth platforms that were available mid-Covid-19 likely impacted care access for families unable to schedule telehealth visits for a variety of reasons, not the least of which may be poor connectivity (63). This may have disproportionately impacted some families and could be associated with some of the observed racial and ethnic disparities in the current study.

Second, clinical staffing should be taken into account when considering service access. Staffing for IPC consultation and co-located services included both faculty/attending psychologists, and supervised predoctoral psychology interns and postdoctoral psychology fellows. For IPC consultation, one psychology provider was staffed per each half-day clinic for only 6-8 out of the 10 half-day clinics per week serving a total of 5 primary care clinics during each half day. This led to some gaps in IPC coverage across the week for both cohorts in the study. So in addition to telehealth specific contextualization, our findings may be especially applicable for populations in clinics where the ratio of BHPs to PCPs is low. For very large clinics, or clinics fully staffed with BHPs, our results may not generalize.

Third, similar to staffing levels for IPC consultation and co-located services, overall behavioral health staffing for SBH services did not vary notably pre-Covid-19 compared to mid-Covid-19. However, across service levels providers may have been out of office more mid-Covid-19 due to illness or to care for ill family members. In this manner, staff coverage during the pandemic may have inadvertently impacted the ability to serve eligible patients and relatedly then impacted the results of this study.

Fourth, our mid-Covid-19 cohort only had scheduled IPC consultation options available to them, which has shown to lead to disproportionate access to care with the warm hand-offs at the point of service, elevating equity issues for IPC access (28, 38). Fifth, while comparisons are made using appropriate statistical analyses, groups were not randomly assigned. Consequently, the design of this study lends itself well to understanding associations between the focal variables but does not allow for causal interpretations of effects, limiting inferences that may be drawn from our results. In addition, there could exist other confounders not measured in this study that could have impacts on the results.

### 4.4 Future directions

A growing body of literature reveals the benefits of embedding behavioral health service support in the places where children and youth spend their time. Evidence suggests that primary care is an ideal and necessary setting to more equitably meet pediatric behavioral health needs. Future directions of this work include increasing the workforce for IPC to facilitate more expedient, equitable, and efficient management of service needs in this setting. This will be more acceptable to families who are already familiar and comfortable with going to their PCMH for care. To increase care access for people of color, and especially Black youth and families, provider-patient concordance in racial identity or language may be important considerations to explore (78–80). Additionally, IPC models have shown to mitigate the challenges of an overburdened SBH care system and sometimes backlogged co-located services that have long waitlists causing patients in need to manage crises for themselves, use emergency services that are not appropriate for the behavioral health concern, or do nothing at all. Finally, while advances in telehealth are appreciated for those who may access services remotely, “One size does not fit all.” Thus, it is essential that we continue to provide in person care and offer both scheduled and ample same-day visit opportunity in primary care as the gold standard for equitable IPC.

### 4.5 In conclusion

The current study involved a retrospective analysis of access to co-located and SBH services for a pre-Covid-19 cohort and a mid-Covid-19 cohort, while accounting for integrated primary care consultation services and sociodemographic factors. The study results showed that (a) the majority of youth were not directly scheduled for co-located or SBH visits but the majority of those scheduled attended their visit(s), (b) the odds of not being directly scheduled for a co-located or SBH visit were greater for the mid-Covid-19 cohort, Black youth, and older youth, and (c) accounting for integrated primary care consultation visits addressed these disparities, with the exception of persisting significant differences in scheduled and attended co-located and SBH visits for Black youth even while accounting for IPC consultation.

This study adds to the current literature in important ways. In particular, it supports continued research on the necessity of culturally grounded strategies to address disparities in behavioral health care access through IPC pathways and provides new evidence to inform this work. These efforts are especially well-situated for primary care where initial pediatric behavioral health help-seeking frequently occurs. The results of this study amplify the need for research and program development that uses tailored strategies to increase access to evidence-based behavioral health services and calls for a continuing shift in behavioral health systems toward advancing equity, including through IPC.

## Data availability statement

The datasets presented in this article are not readily available because they are not publicly available. Requests to access the datasets should be directed to AC, achakawa@cmh.edu.

## Ethics statement

The studies involving humans were approved by Children’s Mercy Institutional Review Board. The studies were conducted in accordance with the local legislation and institutional requirements. Written informed consent for participation was not required from the participants or the participants’ legal guardians/next of kin because retrospective data analyses using medical record data was conducted with no participant contact or interaction.

## Author contributions

AC: Conceptualization, Data curation, Formal analysis, Investigation, Methodology, Project administration, Resources, Validation, Writing – original draft, Writing – review & editing. TC: Conceptualization, Writing – original draft, Writing – review & editing. LB: Writing – original draft, Writing – review & editing. HY: Formal analysis, Writing – review & editing.

## Footnotes

a. ‘Minoritized’ (group) is a term used to describe “ *a community of people whose access to institutional and structural power has been severely limited regardless of the size of the population. As a result, the community is constantly being disenfranchised and disempowered by the majoritized population*.” (81)

b. ‘Integrated Primary Care’ (IPC) is a term used to describe “*a population-based model of care in that it seeks to serve the entire population of a primary care clinic, rather than a small subset of patients with the most significant mental health concerns. To ensure continued access in the system for new patients, treatment is time limited, and stepped care is emphasized. That is, individuals who do not benefit sufficiently from brief treatment can be stepped up to a higher level of care, such as specialty mental health treatment. IPC is also highly collaborative in that behavioral health providers are integrated as part of the care team to provide consultation to medical staff about how to manage behavioral health concerns and coordinate care for behavioral health and physical conditions*.” (82)

c. ‘Communities of color’ (also referred to as ‘people of color’) is a term representing *“a shift from the term “minority” to refer to individuals from diverse racial and ethnic backgrounds. Although it is acceptable for communities to be described as “minoritized,” it is not recommended to refer to people or a population as minority. Instead, use “people of color” or “communities of color” when referring to groups from diverse backgrounds who are non-White. When appropriate, you may use the terms underserved, underrepresented, or marginalized to describe populations of color; however, use the specific group title whenever possible* [e.g., Black or African American, White or European American, Hispanic or Latin/o/e].” (83) Some racial justice advocates recommend using the term BIPOC (which stands for ‘Black, Indigenous, and people of color’) in the U.S. to show solidarity between communities of color and emphasize unique aspects of systemic racism that Black and Indigenous groups experience and the ramifications of slavery and genocide (84).

d. ‘Majoritized’ (group) is a term used to describe “*a community of people whose access to institutional and structural power has been structurally guaranteed, regardless of the size of the population. As a result, the [overall] community [actions] routinely disenfranchise and disempower the most vulnerable communities known as the minoritized populations*.” (81)
